# Transcriptomics data of 11 species of yeast identically grown in rich media and oxidative stress conditions

**DOI:** 10.1186/s13104-019-4286-0

**Published:** 2019-05-03

**Authors:** William R. Blevins, Lucas B. Carey, M. Mar Albà

**Affiliations:** 1 Evolutionary Genomics Group, Research Programme on Biomedical Informatics, Hospital del Mar Research Institute and Universitat Pompeu Fabra, Barcelona, Spain; 2 Single Cell Behavior Group, Department of Experimental and Health Sciences, Universitat Pompeu Fabra, Barcelona, Spain; 3 Center for Quantitative Biology and Peking-Tsinghua Joint Center for Life Sciences, Academy for Advanced Interdisciplinary Studies, Peking University, Beijing, China; 4 Catalan Institution for Research and Advanced Studies, Barcelona, Spain

**Keywords:** RNA-seq, Yeast, Transcriptomics, De novo transcript assembly, De novo gene, Gene annotation

## Abstract

**Objective:**

The objective of this experiment was to identify transcripts in baker’s yeast (*Saccharomyces cerevisiae*) that could have originated from previously non-coding genomic regions, or de novo. We generated this data to be able to compare the transcriptomes of different species of Ascomycota.

**Data description:**

We generated high-depth RNA sequencing data for 11 species of yeast: *Saccharomyces cerevisiae, Saccharomyces paradoxus, Saccharomyces mikatae, Saccharomyces kudriavzevii, Saccharomyces bayanus, Naumovia castelii, Kluyveromyce*s *lactis, Lachancea waltii, Lachancea thermotolerans, Lachancea kluyveri*, and *Schizosaccharomyces pombe*. Using RNA-Seq from yeast grown in rich and oxidative conditions we created genome-guided de novo assemblies of the transcriptomes for each species. We included synthetic spike-in transcripts in each sample to determine the lower limit of detection of the sequencing platform as well as the reliability of our de novo transcriptome assembly pipeline. We subsequently compared the de novo transcripts assemblies to the reference gene annotations and generated assemblies that comprised both annotated and novel transcripts.

## Objective

Due to pervasive transcription and pervasive translation in these yeast, new transcripts and ORFs can quickly appear in non-genic sequences and become exposed to selection. This process, known as de novo gene birth, can lead to the appearance of new genes with entirely novel functions. Our objective was to identify and characterize putative de novo genes in baker’s yeast to further understand the phenomenon of de novo gene birth. To correctly classify putative de novo genes via the taxonomic conservation of these unique sequences, we need comparable data for a set of closely related species. Due to the similarity of molecular pathways to more complex eukaryotes coupled with their ease of growth in the lab, budding yeasts have proved to be a popular group of organisms for experiments ranging from experimental evolution to genetic engineering. We selected these 11 species based on their sparse taxonomic distribution, their amenability to growth in a custom rich media, the availability of genome assemblies, and their inclusion in previous studies of de novo genes in yeast. We have used novel transcripts assembled from our RNA-Seq data, taken together with the reference annotations, to generate a more complete transcriptome for each of the eleven species surveyed. We have estimated the time that each *S. cerevisiae* transcript originated in the yeast phylogeny using homology searches and genomic synteny [1]. As organisms modify their expression and translation of genes in response to stress, we sequenced the transcriptomes of all 11 species of yeast in both rich media and oxidative stress conditions to capture potential transcriptome variability.

The availability of complete gene annotations is key for genome-wide studies. The transcript assemblies provided contain hundreds of transcripts that were not present in the available annotations, and thus provide a more complete view of the gene content of each organism than previous annotations. These transcriptomes can be used as a basis to discover new encoded proteins, to study the evolution of yeast gene families and to investigate the changes in gene expression across different Saccharomycotina species. The addition of the ERCC Spike-into all samples also allows for the benchmarking of different de novo transcriptome assembly protocols.

## Data description

We grew 11 species of yeast in two conditions:

1. *Rich medium* The yeast were grown in 20 mL of a custom rich medium [2], which was shown to accommodate various species of yeast, in 50 mL Erlenmeyer flasks at 30 °C. Cells were harvested in log growth phase at an OD_600_ of approximately 0.25.
2. *Oxidative stress* The same isogenic populations of yeast were grown in parallel, identical to the first condition. However, 30 min prior to harvesting the cells, hydrogen peroxide was added to a final concentration of 1.5 mM; we used a time period of 30 min to maximize the cellular response to stress [3], and a concentration of 1.5 mM H_2_O_2_ as we observed the yeast to grow approximately twice as slowly at this concentration.

After extraction, purification, and polyA selection of the RNA, synthetic spike-in transcripts from the ERCC RNA Spike-in kit [4] were added to each sample in order to assess the performance and limitations of our pipeline. After library preparation, the libraries were pooled into two batches (normal/stress) and sequenced in one lane on the Illumina HiSeq 2500 (paired-end, stranded, 50 bp long). This generated > 20 million high-quality strand-specific read pairs per sample (Table 1).

**Table 1:** Overview of data files

Label	Name of data file/data set	File types (file extension)	Data repository and identifier (DOI or accession number)
*S. cerevisiae* rich medium RNA-seq	SRR8690267_1.fastq; SRR8690267_2.fastq	.fastq	https://identifiers.org/ncbi/insdc.sra:SRR8690267
*S. paradoxus* rich medium RNA-seq	SRR8690268_1.fastq; SRR8690268_2.fastq	.fastq	https://identifiers.org/ncbi/insdc.sra:SRR8690268
*S. mikatae* rich medium RNA-seq	SRR8690269_1.fastq; SRR8690269_2.fastq	.fastq	https://identifiers.org/ncbi/insdc.sra:SRR8690269
*S. kudriavzevii* rich medium RNA-seq	SRR8690270_1.fastq; SRR8690270_2.fastq	.fastq	https://identifiers.org/ncbi/insdc.sra:SRR8690270
*S. bayanus* rich medium RNA-seq	SRR8690271_1.fastq; SRR8690271_2.fastq	.fastq	https://identifiers.org/ncbi/insdc.sra:SRR8690271
*N. castelii* rich medium RNA-seq	SRR8690272_1.fastq; SRR8690272_2.fastq	.fastq	https://identifiers.org/ncbi/insdc.sra:SRR8690272
*K. lactis* rich medium RNA-seq	SRR8690273_1.fastq; SRR8690273_2.fastq	.fastq	https://identifiers.org/ncbi/insdc.sra:SRR8690273
*L. waltii* rich medium RNA-seq	SRR8690274_1.fastq; SRR8690274_2.fastq	.fastq	https://identifiers.org/ncbi/insdc.sra:SRR8690274
*L. waltii* replicate 2 rich medium RNA-seq	SRR8690275_1.fastq; SRR8690275_2.fastq	.fastq	https://identifiers.org/ncbi/insdc.sra:SRR8690275
*L. thermotolerans* rich medium RNA-seq	SRR8690276_1.fastq; SRR8690276_2.fastq	.fastq	https://identifiers.org/ncbi/insdc.sra:SRR8690276
*L. kluyveri* rich medium RNA-seq	SRR8690261_1.fastq; SRR8690261_2.fastq	.fastq	https://identifiers.org/ncbi/insdc.sra:SRR8690261
*Schizo. pombe* rich medium RNA-seq	SRR8690262_1.fastq; SRR8690262_2.fastq	.fastq	https://identifiers.org/ncbi/insdc.sra:SRR8690262
*S. cerevisiae* oxidative stress RNA-seq	SRR8690267_1.fastq; SRR8690267_2.fastq	.fastq	https://identifiers.org/ncbi/insdc.sra:SRR8690267
*S. paradoxus* oxidative stress RNA-seq	SRR8690268_1.fastq; SRR8690268_2.fastq	.fastq	https://identifiers.org/ncbi/insdc.sra:SRR8690268
*S. mikatae* oxidative stress RNA-seq	SRR8690257_1.fastq; SRR8690257_2.fastq	.fastq	https://identifiers.org/ncbi/insdc.sra:SRR8690257
*S. kudriavzevii* oxidative stress RNA-seq	SRR8690258_1.fastq; SRR8690258_2.fastq	.fastq	https://identifiers.org/ncbi/insdc.sra:SRR8690258
*S. bayanus* oxidative stress RNA-seq	SRR8690259_1.fastq; SRR8690259_2.fastq	.fastq	https://identifiers.org/ncbi/insdc.sra:SRR8690259
*N. castelii* oxidative stress RNA-seq	SRR8690260_1.fastq; SRR8690260_2.fastq	.fastq	https://identifiers.org/ncbi/insdc.sra:SRR8690260
*K. lactis* oxidative stress RNA-seq	SRR8690265_1.fastq; SRR8690265_2.fastq	.fastq	https://identifiers.org/ncbi/insdc.sra:SRR8690265
*L. waltii* oxidative stress RNA-seq	SRR8690266_1.fastq; SRR8690266_2.fastq	.fastq	https://identifiers.org/ncbi/insdc.sra:SRR8690266
*L. waltii* replicate 2 oxidative stress RNA-seq	SRR8690278_1.fastq; SRR8690278_2.fastq	.fastq	https://identifiers.org/ncbi/insdc.sra:SRR8690278
*L. thermotolerans* oxidative stress RNA-seq	SRR8690277_1.fastq; SRR8690277_2.fastq	.fastq	https://identifiers.org/ncbi/insdc.sra:SRR8690277
*L. kluyveri* oxidative stress RNA-seq	SRR8690280_1.fastq; SRR8690280_2.fastq	.fastq	https://identifiers.org/ncbi/insdc.sra:SRR8690280
*Schizo. pombe* oxidative stress RNA-seq	SRR8690279_1.fastq; SRR8690279_2.fastq	.fastq	https://identifiers.org/ncbi/insdc.sra:SRR8690279
*S. cerevisiae* transcriptome assembly	s_cerevisiae_annotations_with_novel.gff	.gff	10.6084/m9.figshare.7851521.v2
*S. paradoxus* transcriptome assembly	s_paradoxus_annotations_with_novel.gff	.gff	10.6084/m9.figshare.7851521.v2
*S. mikatae* transcriptome assembly	s_mikatae_annotations_with_novel.gff	.gff	10.6084/m9.figshare.7851521.v2
*S. kudriavzevii* transcriptome assembly	s_kudriavzevii_annotations_with_novel.gff	.gff	10.6084/m9.figshare.7851521.v2
*S. bayanus* rich medium RNA-seq	s_bayanus_annotations_with_novel.gff	.gff	10.6084/m9.figshare.7851521.v2
*N. castelii* transcriptome assembly	n_castellii_annotations_with_novel.gff	.gff	10.6084/m9.figshare.7851521.v2
*K. lactis* transcriptome assembly	k_lactis_annotations_with_novel.gff	.gff	10.6084/m9.figshare.7851521.v2
*L. waltii* transcriptome assembly	l_waltii_annotations_with_novel.gff	.gff	10.6084/m9.figshare.7851521.v2
*L. waltii* replicate 2 transcriptome assembly	l_waltii_rep2_annotations_with_novel.gff	.gff	10.6084/m9.figshare.7851521.v2
*L. thermotolerans* transcriptome assembly	l_thermotolerans_annotations_with_novel.gff	.gff	10.6084/m9.figshare.7851521.v2
*L. kluyveri* transcriptome assembly	l_kluyveri_annotations_with_novel.gff	.gff	10.6084/m9.figshare.7851521.v2
*Schizo. pombe* transcriptome assembly	schizo_pombe_annotations_with_novel.gff	.gff	10.6084/m9.figshare.7851521.v2
Sources of yeast strains and reference genomes	Yeast_strain_source.pdf	.pdf	10.6084/m9.figshare.7851521.v2

The raw RNA-Seq data (.fastq files) are available for download on the SRA [7], and the transcriptome assembly annotations (.gff files), consisting of novel transcripts combined with the reference annotations for each species, are available on Figshare [8] as well as a table with the information about the source of each strain and reference genome

After taking some quality control measures with our raw RNA-Seq data, we mapped the reads to their respective genomes (Table 1) and assembled de novo transcriptomes using the program Trinity version 2.1.0 [5]. We created a non-redundant set of features from the reference annotations combined with our de novo assembled transcripts; de novo assembled transcripts which correspond to annotated features according to Cuffmerge version 2.2.0 [6] were discarded, while those that did not were considered to be novel; we identified an average of 700 novel transcripts per species [1] (Table 1). The majority of these novel transcripts were found to be expressed in both conditions, but dozens of transcripts were only expressed in one condition or the other. Using the ERCC RNA Spike-in [4], we calculated that the lower limit of detection for annotated features in our pipeline was 2 TPM, and the lower limit of expression necessary to reliably assemble novel transcripts was 15 TPM; over half of the unannotated transcripts that we assembled were expressed above this conservative threshold of 15 TPM in at least one of the two conditions.

## Limitations

A limitation of this dataset is that there are not multiple replicates for each species/condition, except for *L. waltii,* which has two replicates in each condition. We also would like to acknowledge that the concentration of hydrogen peroxide we used to induce an oxidative stress response (1.5 mM) was higher than the concentration used in other studies of oxidative stress response in yeast (0.1–1 mM).

## Abbreviations

RNA-Seq: RNA sequencing
TPM: transcripts per million, a normalized measure of mRNA abundance
ERCC: External RNA Control Consortium
mM: millimolar, a measure of concentration
H_2_O_2_: hydrogen peroxide
